# Efficacy of intra-arterial chemotherapy with sequential anti-PD-1 antibody in unresectable gastric cancer: A retrospective real-world study

**DOI:** 10.3389/fonc.2022.1015962

**Published:** 2023-01-05

**Authors:** Xiaosong Xiang, Feilong Guo, Guoli Li, Long Ma, Xi Zhu, Zulpikar Abdulla, Jiafei Li, Junling Zhang, Mengli Huang

**Affiliations:** ^1^ Department of General Surgery, Jinling Hospital, School of Medicine, Nanjing University, Nanjing, China; ^2^ Department of Biostatistics, Columbia University, New York, NY, United States; ^3^ The Medical Department, 3D Medicines Inc., Shanghai, China

**Keywords:** conversion therapy, unresectable gastric cancer, anti-PD-1 antibody, intra-arterial chemotherapy, immune-based combination therapies

## Abstract

**Background:**

The prognosis of unresectable gastric cancer is poor, while the efficacy of anti-PD antibodies has not been evaluated.

**Methods:**

Patients with unresectable gastric cancer who received intra-arterial chemotherapy (IAC) with sequential anti-PD-1 antibody as induction therapy in Jinling Hospital were retrospectively analyzed. The primary outcome is R0 resection rate. The secondary outcomes include safety, conversion surgery rate, overall survival (OS) and progression free survival (PFS) after postoperative IAC and anti-PD-1 treatments. Meanwhile, Tumor immunity in the microenvironment (TIME) before and after IAC was comprehensively dissected with multiplex immunofluorescence in order to detect possible mechanisms favoring anti-PD-1 treatment response.

**Results:**

Between May 2019 and October 2020, 36 patients received at least one cycle of IAC with sequential anti-PD-1 antibody in our institution. The objective response was achieved in 28 patients (77.8%). Thirty patients (83.3%) successfully underwent conversion surgery, among which R0 resection was managed in 25/30 patients, and 23.3% (7/30) was assessed as pathological complete remission. During the median follow-up period of 19.7 months, patients who underwent R0 resection displayed superior OS (HR 0.14 [95% CI 0.04-0.50], P < 0.0001) and PFS (HR 0.11 [0.03-0.44], P < 0.0001) than those who did not. Grade 3 adverse events (AEs) were only encountered in 19.4% patients, no grade 4 AEs observed. In TIME analysis, the number of tertiary lymphoid structures (TLSs) (P = 0.004) were greatly induced by IAC, as well as CD8^+^ T cells (P = 0.011) and PD-1^+^ cells (P = 0.025). Meanwhile, Tumor associated macrophages shifted towards anti-tumor M1-like subtypes, with CD68^+^CD163^+^ M2-like subpopulation significantly decreased (P = 0.04).

**Conclusion:**

Preoperative IAC with sequential anti-PD-1 antibody exhibited promising clinical benefit for unresectable gastric cancer with remarkable conversion rate and R0 resection rate, and also prolonged survival as postoperative regimen. TIME transformation induced by ICA might mediate the additive effect with the immune checkpoint inhibitor.

## Introduction

Despite improvements in early screening and intensive therapy, gastric cancer (GC) is still the fourth leading cause of cancer-related death in the world according to GOLBOCAN 2020 (1). The survival of patients with unresectable conditions, either technically or oncologically, such as adjacent organs invasion, vital blood vessels encompassing, peritoneal dissemination, distant lymph node/organ metastasis, etc., was especially poor. Systemic chemotherapy is currently the standard treatment (2, 3), but the median survival reported was usually around one year (4–6).

The REGATTA trial revealed that non radical resection first plus adjuvant chemotherapy could not provide superior overall survival to chemotherapy alone in initially unresectable patients (7). However, several previous studies indicated patients who well responded to preoperative induction therapy and were managed to undergo radical therapy could achieve remarkable long-term survival benefit (8–10). Thus, conversion therapy which refers to the treatment strategy aimed at R0 resection after chemotherapy or other comprehensive therapeutic methods to reduce the volume or stage of the tumors that were previously impossible or difficult to be radically resected, has attracted much attention in recent years for unresectable gastric cancer (11, 12). Unfortunately, the research on conversion therapy based on the existing systematic chemotherapy regimens has not made a breakthrough, both the conversion surgery rate and R0 resection rate were unsatisfactory (11, 13–17), therefore new induction regimens were being expected.

Immunotherapy represented by anti-PD antibodies has displayed excellent efficacy in the treatment of various tumors, and the clinical efficacy in advanced GC has also been well documented (18, 19), which sheds new light on the conversion therapy for GC. On the other hand, these studies also indicated that only a small subset of GC patients could benefit from anti-PD therapies (20), conversely many exhibited primary resistance with diverse and complex mechanisms to be explored, among which tumor immunity in the microenvironment (TIME) was considered to play the pivotal role (21). Current studies of TIME mainly focused on the tumor infiltrating leukocytes (TILs) and the expression of PD-L1/PD pathway. Infiltration of CD8^+^ effector T cells or high expression of PD-L1 was detected to underlie a better treatment response (22–24). Unfortunately, TIME of most advanced GC patients belonged to the immunological ignorant or resistant type, characterized with TILs insufficiency and/or lack of PD-L1 expression (20, 25). Therefore, TIME modulation, making it more suitable for anti-PD therapies, emerges as a rational treatment strategy, and combination treatment or sequential treatment has been proposed (21).

Intra-arterial chemotherapy, which administrated concentration-dependent cytotoxic drugs like oxaliplatin directly into the primary tumor site *via* tumor supplying vessels (usually the left gastric artery or the right gastroepiploic artery) and initiated in our institution since 2002, has demonstrated excellent efficacy and superior clinical outcomes in locally advanced or advanced GC patients (26–28). Different from systemic intravenous infusion, high concentration of oxaliplatin concentrated in tumor site was prone to invoke immunogenic tumor cell death, then subsequent tumor antigen presentation and priming of effector T cells (29), which should be an efficient TIME modulating method and might synergize with the sequential anti-PD therapies theoretically. Therefore, we speculated that intra-arterial chemotherapy with sequential anti-PD antibody would be a feasible conversion therapy for patients with unresectable GC, and TIME transformation induced by intra-arterial chemotherapy might mediate the synergistic effect.

As such, we conducted the real-world study to evaluate the clinical effect and safety of this new regimen: intra-arterial chemotherapy with sequential anti-PD-1 monoclonal antibody in conversion therapy for patients with unresectable GC, and to dissect the alteration of TIME pro- and post- intra-arterial chemotherapy in order to detect possible scientific basis underlying the combination treatment.

## Methods

### Patients for conversion therapy

Patients with unresectable gastric adenocarcinoma who received intra-arterial chemotherapy plus anti-PD-1 antibody were recorded in our prospectively maintained Gastric Cancer database. The diagnosis of gastric adenocarcinoma was histologically confirmed by endoscopic biopsy. Unresectable conditions were determined by our multidisciplinary team mainly based on contrast-enhanced computed tomography (CT), magnetic resonance imaging (MRI) and staging laparoscopy if peritoneal metastasis suspected. Unresectable factors could be summarized as the following categories: (1) bulky lymph nodes encompassing vital blood vessels, (2) large distal tumor affecting primary closure of duodenum stump, (3) tumor invasion of adjacent organs, (4) peritoneal metastasis including macroscopic peritoneal dissemination and positive peritoneal lavage cytology, (5) para-aortic, left supraclavicular, or other distant lymph node metastasis, (6) distant organ metastasis including liver metastasis, Krukenberg tumor, and etc.

Intra-arterial chemotherapy with sequential anti-PD-1 antibody started from May, 2019 in patients who provided written informed consent in our institution. Additional inclusion criteria were: (1) age 18 years and older, (2) Eastern Cooperative Oncology Group (ECOG) performance status of 0-2, (3) well-preserved function of main organs, (4) no prior radiotherapy, chemotherapy or any other targeted therapies for the current gastric cancer, (5) no history of gastrectomy, (6) no history of gastrointestinal malignancies, (7) at least one measurable lesion per Response Evaluation Criteria in Solid Tumors (RECIST) guidelines (version 1.1). Patients’ demographic information, tumor characteristics, therapeutic and survival data were all extracted from the prospectively maintained database. All the procedures were approved by the Institutional Review Board of Jinling Hospital (2022DZSKT-039).

From May 2019 to October 2020, 36 consecutive patients diagnosed with unresectable gastric adenocarcinoma received intra-arterial chemotherapy with sequential anti-PD-1 antibody in our institution. Baseline characteristics and demographics are summarized in **Table 1**. The mean age of these patients was 61 years old, and the male dominated over 80% of all cases. Among these patients, Bulky lymph nodes encompassing vital blood vessels were confirmed in 14 patients. Large distal tumor affecting the closure of duodenal stump was identified in 5 patients. Adjacent organ invasion was detected in 5 patients, with diaphragm involvement in three, and transverse colon in two. Peritoneal lavage cytology was positive in 4 patients. Distant lymph node metastasis was observed in 14 patients, with para-aortic lymph node metastasis in thirteen, and left clavicle lymph node metastasis in one. Distant organ metastasis occurred in 4 patients, with liver metastasis in two, ovary metastasis in one, and descending colon metastasis in one. Of note, 10 patients concomitantly possessed two unresectable factors. Detailed unresectable conditions of each patient were summarized in the **Supplementary Table S1**.

**Table 1:** Patient demographics and tumor characteristics.

Variable	Patients (N=36)
Age (years), mean (range)	61.4 (29–74)
Sex, N (%)	
Male	29 (80.6%)
Female	7 (19.4%)
Tumor Location, N (%)	
Upper third	11 (30.6%)
Lower third	16 (44.4%)
Diffuse or undefined	9 (25%)
Borrmann Type, N (%)	
I	1 (2.8%)
II	3 (8.3%)
III	10 (27.8%)
Large III(> 8 cm)	8 (22.2%)
IV	14 (38.9%)
Tumor invasion^*^, N (%)	
cT3	2 (5.6%)
cT4a	23 (63.9%)
cT4b	11 (30.6%)
Lymph node metastasis^*^, N (%)	
cN1	6 (16.7%)
cN2	3 (8.3%)
cN3	27 (75%)
TNM stage^*^, N (%)	
cIVa	16 (11.1%)
cIVb	20 (55.6%)
Unresectable or marginally radical resection reason, N (%)	
Bulky lymph node encompassing vital blood vessels	14 (38.9%)
Large distal tumor affecting duodenum closure	5 (13.9%)
Tumor invasion of adjacent organs	5 (13.9%)
Peritoneal metastasis	4 (11.1%)
Distant lymph node metastasis	14 (38.9%)
Distant organ metastasis	4 (11.1%)

^*^ In accordance with the AJCC eight edition gastric cancer TNM staging system.

### Treatment procedures

Enrolled participants received the combination of intra-arterial chemotherapy and anti-PD-1 antibody every 3 weeks for 3 cycles. The regimens of intra-arterial chemotherapy in our institution were described in the previous study in detail (28). Briefly, oxaliplatin (100 mg/m^2^), etoposide (100mg/m^2^), and epirubicin (30 mg/m^2^) were intraarterially administrated to tumor supplying vessels *via* femoral artery cannulation with Seldinger method on day 1, usually super-selecting the left gastric artery for tumors in the upper and middle stomach, while the right gastroepiploic artery for tumors in the lower stomach. S-1 (40-60 mg twice daily depending on body surface area (BSA), 40mg for BSA < 1.25 m^2^, 50mg for BSA 1.25-1.5 m^2^, 60mg for BSA > 1.5 m^2^) was given orally on days 1-14, with one week rest [SEEOX]. In addition, 200 mg anti-PD-1 monoclonal antibody (Sintilimab, Innovent Biologics Co Ltd, Suzhou, China) was injected intravenously on day 2 at each cycle. Dose reduction, delay, or cessation was prescribed according to adverse events (AEs). The immunotherapy was immediately terminated once tumor hyperprogression was detected.

Tumor response was evaluated according to the RECIST guidelines (version 1.1) with CT or MRI at baseline, then every two cycles and 7-10 days after the last cycle. But extra evaluation was added if patients’ symptoms worsened and persisted. The response included complete response (CR), partial response (PR), stable disease (SD) and progressive disease (PD). AEs were assessed continuously during the whole induction therapy period based on the National Cancer Institute Common Terminology Criteria for Adverse Events (version 4.0).

Laparotomy with/without re-peritoneal lavage cytology was suggested in patients who achieved CR and PR after the last treatment, and also performed in patients who remained SD and were willing to accept the abdominal exploration. If unresectable conditions were ameliorated and R0 resection became possible, gastrotomy with radical lymphadenectomy, plus combined organ resection (if needed according to the intraoperative condition) as conversion surgery were performed. Otherwise, palliative operation or direct abdominal closure was applied. For patients who suffered from PD or refused surgical treatment, palliative chemotherapy or best care support took over.

Surgical complications were assessed by Clavien-Dindo classification. Histology response in the primary tumor was graded according to TRG classification, which included the following four grades: grade 0, complete response, no viable tumor cells remained including the lymph nodes; grade 1, moderate response, single cells or small group of cancer cells; grade 2, minimal response, residual cancer outgrown by fibrosis; grade 3, poor response, minimum of no treatment effect, extensive residual cancer cells. Patients with TRG 0-2 were considered pathological responders.

Postoperative chemotherapy was initiated about 4 weeks after surgery. Patients with R0 resection received at least 3 cycles of adjuvant chemotherapy. The basic regimen was oxaliplatin (intravenous infusion of 100 mg/m^2^ on day 1) plus S-1 (the same dosage and duration with preoperative chemotherapy) [SOX] every 3 weeks. Those who wished to continue with immunotherapy, 200mg PD-1 antibody (the same brand with that used before surgery) was intravenously added on day 2 in each cycle. For patients with R1 resection, SOX ± PD-1 was applied for at least 3 cycles, and S-1 monotherapy was continued until progression was identified. For patients without radical resection, palliative chemotherapy was also proposed after surgery.

### Outcome measurement

The primary outcome is R0 resection rate. The secondary outcomes include treatment safety, objective response rate (ORR), conversion surgery rate, overall survival (OS) and progression-free survival (PFS). ORR was defined as the proportion of patients who achieved CR or PR in all participants enrolled in conversion therapy. Macroscopically radical operation (R0 plus R1) without perioperative death was considered successful conversion surgery. The OS period was defined as the time from the initiation of the preoperative induction therapy to death, and the PFS was defined as the period from the initiation of the preoperative induction therapy to tumor progression or death, whichever came first.

### Patients for TIME evaluation

In addition, the study explored TIME alteration before and after intraarterial chemotherapy in 7 patients who were histologically confirmed gastric adenocarcinoma and received SEEOX as neoadjuvant chemotherapy in our institution. The inclusion criteria were: (1) primary gastric adenocarcinoma without any chemotherapy, immunotherapy or targeted therapy before; (2) clinical TNM stage was T3-4a N+ M0 or T4b Nany M0. The protocol of SEEOX in these patients was the same with that described above, and all patients received 3 cycles of neoadjuvant chemotherapy before surgery. Preoperative specimen naïve to any treatment obtained by endoscopy and surgical specimen after 3 cycles of SEEOX of the same patient were both preserved for further TIME analysis.

### TIME analysis

Formalin-fixed paraffin-embedded tumor tissue sections were prepared. Samples were stained using an Opal automation mIF detection kit (Akoya, Tokyo, Japan). The following antibodies were used: anti-CD163 (Abcam, Cat#ab182422), anti-CD8 (Abcam, Cat# ab178089), anti-CD68 (Abcam, Cat#ab213363), anti-PD-1 (Cell Signaling Technology, Cat#86183S), anti-PD-L1 (Cell Signaling Technology, Cat#13684S), and anti-panCK (Abcam, Cat#ab7753), CD20 (Daco, Cat#L26 IR604), CD3 (Daco, Cat#A0452 IR503), CD56 (Abcam, Cat#ab75813), CD4 (Abcam, Cat# ab133616), FoxP3 (Abcam, Cat# ab20034), and panCK. The Vectra Polaris Automated Quantitative Pathological Imaging System (Akoya) was used to overlay false colors on images from different channels. Tumor and stroma areas were divided according to cytokeratin (CK)-labeled tumor cells and nuclei stained with 4′-6′-diamidino-2-phenylindole (DAPI). Results are reported as percentages (immune subset cells/total cells of DAPI).

### Statistical analysis

Continuous variables were expressed as median or mean with range and compared using Mann-Whitney U test. Categorical variables were compared using Chi-square or Fisher exact test. The OS and PFS were calculated with the Kaplan-Meier method and compared using log-rank test. For all analyzes, p value <0.05 was considered to be statistically significant. Statistical analyses were performed using IBM SPSS Statistics 22.0 (IBM Corp., Armonk NY, USA).

## Results

### Preoperative chemotherapy, clinical response and conversion surgery

**Figure 1:**
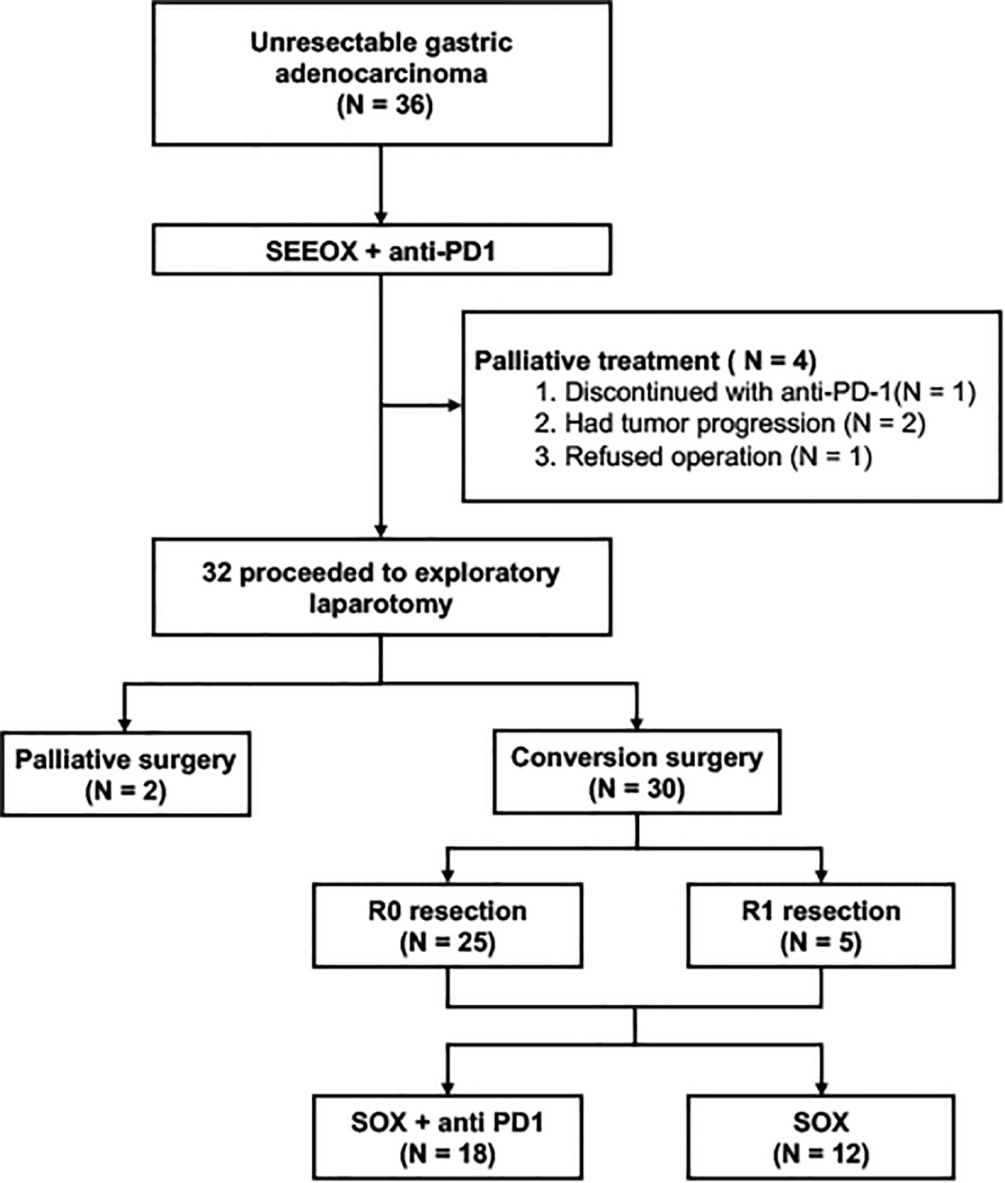
Study profile of the conversion therapy. SEEOX, S-1, etoposide, epirubicin and oxaliplatin. SOX, S-1 and oxaliplatin. PD-1, programmed cell death-1.

**Figure 2:**
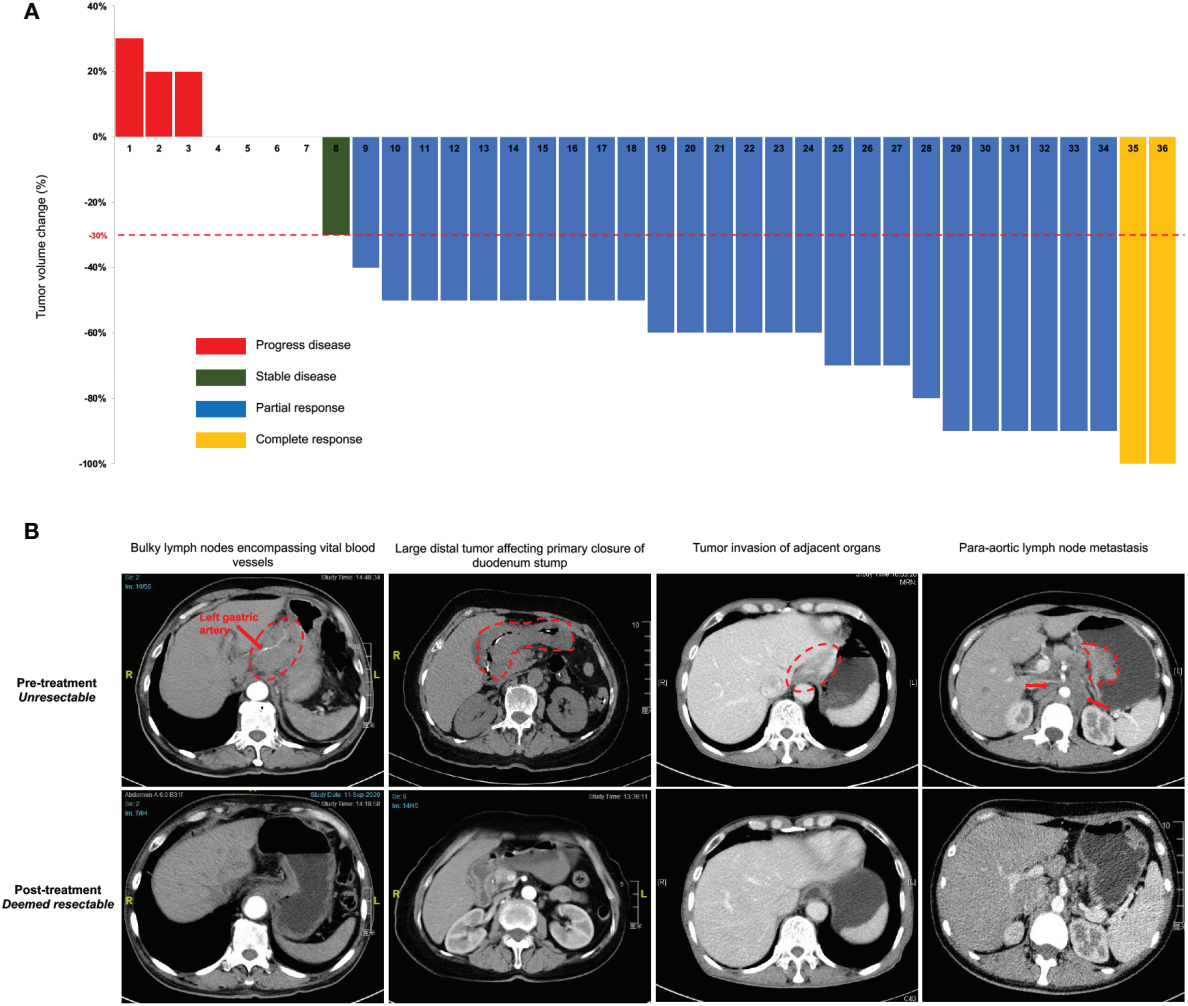
Clinical response to intra-arterial chemotherapy with sequential anti-PD-1 antibody. **(A)** Tumor volume changes from the baseline per Response Evaluation Criteria in Solid Tumors (RECIST) guidelines (version 1.1) in all enrolled 36 patients after the last induction treatment. **(B)** Representative computed tomography images illustrate several pre-treatment unresectable conditions and post-treatment improvement. The red dash circle and arrows indicate corresponding unresectable factors. Two images in the same column are from the same patient before and after the induction treatment.

Thirty-five patients completed the entire 3 courses of SEEOX plus anti-PD-1 antibody with no dose reduction. One patient suffered from para-aortic lymph nodes enlargement after two induction cycles and discontinued anti-PD-1 therapy onwards (**Figure 1**). According to RESISCT 1.1, another 2 patients encountered progression after the last cycle, while CR and PR was respectively achieved in 2 (2/36, 6%) and 26 (26/36, 72%) patients, and the other five patients remained SD (**Figure 2A**). Thus, the ORR reached 77.8%, and the disease control rate was 91.7% in total.

Thirty-three patients without progression were suggested to receive laparotomy or re-laparoscopy, but one of the PR patients refused and continued with palliative chemotherapy. Therefore, a total of 32 patients underwent laparotomy. Pre- and post-treatment representative CT images of these patients were displayed in **Figure 2B**. Radical resection was deemed possible in 30 patients, then D2 or D2+ lymphadenectomy were successfully performed, with total gastrectomy in 18 patients and distal gastrectomy in 12 patients. Meanwhile, one patient with bilateral adnexal metastasis underwent combined bilateral adnexal resection, one patient with splenic hilar lymph node metastasis received combined splenectomy, and 2 patients underwent combined partial colon resection. Consequently, R0 resection was succeeded in 25 patients, while the other 5 patients achieved R1 resection (**Table 2**). Overall, the conversion rate was 83.3% (30/36, 95% CI 71.2%-95.5%), and the R0 resection rate was 69.4% (25/36, 95% CI 54.4%-84.5%). In addition, peritoneal metastasis was detected in one patient and palliative gastrojejunostomy was performed. Descending colon metastases was found difficult to resect in another, thus palliative terminal ileostomy was applied (**Figure 1**).

**Table 2:** Surgical and pathological details.

Variables	Patients (N=30)
Type of resection, N	
Total gastrectomy	18
Distal gastrectomy	12
Combined organ resection, N	
Bilateral adnexectomy	1
Partial colon resection	2
Splenectomy	1
Resection margin, N	
R0	25
R1	5
ypT stage^*^, N	
T0	7
T1b	3
T2	5
T3	3
T4a	9
T4b	3
ypN stage^*^, N	
N0	14
N1	9
N2	4
N3a	2
N3b	1
ypTNM stage^*^, N	
I	9
IIIA	3
IIIB	8
IVA	8
IVB	2
TRG, N	
TRG0	7
TRG1	8
TRG2	4
TRG3	11

^*^ In accordance with the AJCC eight edition gastric cancer TNM staging system.

Histology response was assessed in 30 patients undergoing conversion surgery. The number of patients in grades 0, 1, 2, and 3 were 7, 8, 4 and 11 respectively (**Table 2**). Therefore, the rate of pathological complete remission (TRG0) was achieved in 23.3% (7/30, 95% CI 8.2%-38.5%) of the patients, and the rate of pathological response (TRG0-2) was 63.3% (19/30, 95% CI 46.1%-80.6%). In addition, ypN0 was diagnosed in 14 patients (46.7%, 95% CI 28.8%-64.5%), while ypN3a or ypN3b was only found in 3 patients (10%, 95% CI -0.7%-20.7%; **Table 2**) in accordance with the AJCC eight edition gastric cancer TNM staging system.

Following conversion surgery, 18 patients chose to receive adjuvant therapy of SOX combined with anti-PD-1 antibody, while another 12 patients chose only the first-line adjuvant chemotherapy regimens of SOX (**Figure 1**).

### Short-term survival outcomes

**Figure 3:**
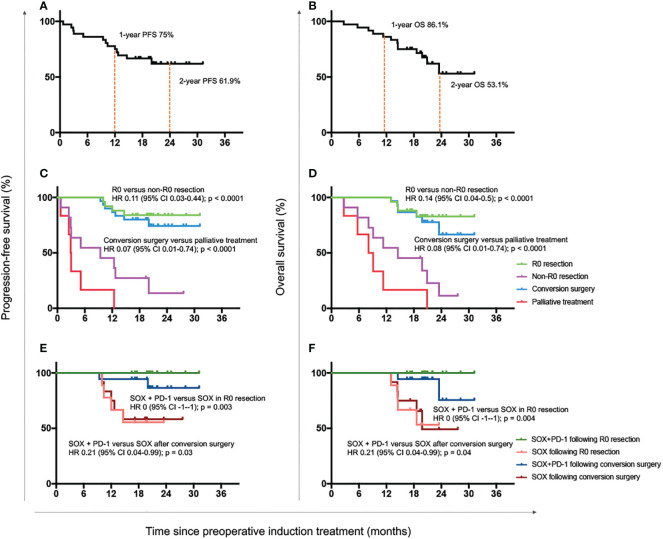
Progression-free and overall survival outcomes. Progression-free survival **(A)** and overall survival **(B)** of all enrolled 36 patients. Progression-free survival **(C)** and overall survival **(D)** of patients with R0 resection, non-R0 resection, conversion surgery and palliative treatment. Progression-free survival **(E)** and overall survival **(F)** of patients who received with the combination of SOX plus anti-PD-1 antibody or only the standard adjuvant chemotherapy regimen SOX after R0 resection or conversion surgery. OS, overall survival; HR, hazard ratio; CI, confidence interval; SOX, S-1 and oxaliplatin; PD-1, programmed cell death-1.

Up to March 2022, the median follow-up duration was 19.7 months (range, 3-31 months). A total of 13 patients died, consisting of all the 6 patients who received no surgery or only palliative surgery. The median survival time of all 36 patients was not reached. The 1- and 2-year OS was 86.1% and 53.1%, while the 1- and 2-year PFS was 75% and 61.9% (**Figures 3A, B**).

The median survival time of patients with R0 resection was also not reached. The 1- and 2-year OS was 100% and 82.8%, and the 1- and 2-year PFS was 88% and 84%. Both the OS and PFS of patients with R0 resection were significantly longer than those with R1 resection or palliative treatment (OS: HR 0.14 [95% CI 0.04-0.50], P < 0.0001; PFS: HR 0.11 [0.03-0.44], P < 0.0001; **Figures 3C, D**). Meanwhile, patients undergoing conversion surgery also had superior OS and PFS to those receiving only palliative treatment (OS: HR 0.08 [95% CI 0.01-0.74], P < 0.0001; PFS: HR 0.07 [0.01-0.74], P < 0.0001; **Figures 3C, D**).

We further detected the survival benefit of postoperative anti-PD-1 treatment. As shown in **Figures 3E, F**, the OS and PFS were both significantly better in patients who continued with anti-PD-1 antibody than those with standard SOX regimens after R0 resection (OS: HR 0 [-1–1], P = 0.004; PFS: HR 0 [-1–1], P = 0.003) or conversion surgery (OS: HR 0.21 [0.04-0.99], P = 0.04; PFS: HR 0.21 [0.04-0.99], P = 0.03).

### AEs and surgical complications

The types and frequencies of AEs encountered during chemotherapy and immunotherapy, as well as surgical complications, were recorded in **Table 3**. 27 (75%) out of 36 patients experienced AEs of any grade. The major AEs include leucopenia, anemia, nausea, vomiting, and diarrhea, grade 3 AEs in 19.4% patients, but no grade 4 AEs were observed. Concerning immunotherapy, we only detected 1 case of dermatitis and 1 case of diarrhea. Autoimmune pneumonia/myocarditis was not reported in the study. Surgical complications were assessed in all 32 patients. Surgical complications included anastomotic fistula in 2 cases and hemorrhage in 1 case. There was no operative mortality or reoperation in this cohort, thus the operative incidence was 9.4% (3/32) in total.

**Table 3:** Summary of adverse events and surgical complications.

Adverse events	Grade 1, N (%)	Grade 2, N (%)	Grade 3, N (%)	Grade 4, N (%)
Chemotherapy				
Leukopenia	9 (25%)	9 (25%)	1 (3%)	0
Anemia	2 (6%)	5 (14%)	2 (6%)	0
Thrombocytopenia	8 (22%)	5 (14%)	2 (6%)	0
Blood bilirubin increased	3 (8%)	0	0	0
AST or ALT increased	6 (17%)	0	0	0
Vomiting	8 (22%)	v3 (8%)	1 (3%)	0
Anorexia	4 (11%)	2 (6%)	0	0
Diarrhea	4 (11%)	1 (3%)	1 (3%)	0
Stomatitis	6 (17%)	1 (3%)	0	0
Fatigue	3 (8%)	1 (3%)	0	0
Immunotherapy				
Dermatitis	1 (3%)	0	0	0
Arthritis	0	0	0	0
Diarrhea/colitis	1 (3%)	0	0	0
Hypothyroidism	0	0	0	0
Autoimmune pneumonia	0	0	0	0
Autoimmune myocarditis	0	0	0	0
Surgical complications				
Anastomotic fistula	0	2 (6%)	0	0
Hemorrhage	0	1 (3%)	0	0
Wound infection	0	0	0	0

AST, aspartate aminotransferase; ALT, alanine aminotransferase.

### Alteration of TIME induced by intra-arterial chemotherapy

In order to explore the effect of intra-arterial chemotherapy on the TIME of GC patients, and to detect possible mechanism underlying the additive effect of the combined therapy, we compared the changes of TIME before and after the intra-arterial chemotherapy (SEEOX) in 7 patients using the multiple immunofluorescence labeling technique. The demographic, oncological and therapeutic information of these patients were summarized on the **supplementary table S2**. Here we mainly reported the number and phenotype of T cells, B cells, tumor-associated macrophages (TAMs), natural killer cells (NKs) and other cells that play important roles in tumor immunity, and also compared the expression of PD-1 in TIME before and after treatment.

**Figure 4:**
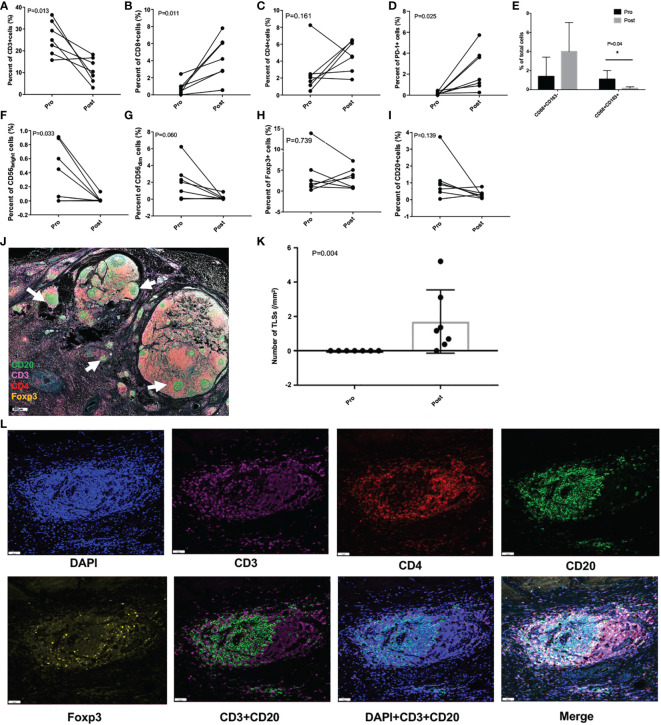
Tumor immunity in the microenvironment pre- and post- intra-arterial chemotherapy. **(A-I)** Per cent changes of different immune cells before and after treatment. **(J)** Representative immunofluorescence image of resected tumor specimen after treatment, and the white arrows indicate the tertiary lymphoid structures (TLSs; scale: 500μm). **(K)** The number of TLSs before and after the treatment. **(L)** Images of representative TLSs (scale: 50μm) *P < 0.05.

As shown in **Figure 4**, CD3^+^ T cells were greatly decreased after treatment (P = 0.013), however CD8^+^ T cells were significantly increased (P = 0.011), and the same increasement was observed in PD-1^+^ cells (P = 0.025). TAM1 and TAM2 cells displayed opposite changing trend, with the percentage of TAM1substantially increased but didn’t reach statistically significance (P = 0.08), while TAM2 remarkably decreased (P = 0.04). The percent of CD56_bright_ cells was also greatly decreased (P = 0.033), but the percent of CD56_dim_ cells showed no significant difference, neither were CD4^+^ T cells, Foxp3^+^ cells, and CD20^+^ cells before and after intra-arterial chemotherapy.

Astonishingly, a large number of tertiary lymphoid structures (TLSs) in the TIME were greatly induced (P = 0.004) after intra-arterial chemotherapy in all these patients, which were rarely found before treatment (**Figures 4J–L**).

## Discussion

To be best of our knowledge, this is the first study exploring the efficacy of anti-PD-1 antibody as the induction regimen followed by conversion surgery for unresectable gastric cancer. To keep consistent and for a better understanding, we continued to use ‘conversion therapy’ when the immunotherapy was added in the setting. As shown above, conversion surgery was managed in 83.3% patients (30/36), and R0 resection rate reached 69.4% (25/36), each of which is superior to previous reported outcomes (11, 13–17, 30). 5-fluorouracil-, platin- and paclitaxel-based doublet or triplet chemotherapies were the most common induction regimens in previous studies (12), conversion surgery was usually applicable in less than 50% patients, while R0 resection was successful in no more than 30% patients (11, 13–17, 30). In two very recent studies, Xu et al (31) and Ye et al (32) added apatinib to conventional systematic chemotherapies and investigated clinical effects, though ORR increased to around 70%, both the conversion surgery and R0 resection rate was still less than 60%. Therefore, intra-arterial chemotherapy (SEEOX) with sequential anti-PD-1 antibody was a promising induction regimen for unresectable gastric cancer.

In recently years, anti-PD therapies have shown encouraging clinical benefit in several malignancies, and favorable outcomes were also observed in advanced gastric cancer. Nivolumab was approved as the third-line or later-line treatment based on ATTRACTION-2 results (33) and pembrolizumab was suggested for microsatellite instability-high (MSI-H) or combined PD-L1 positive score (CPS) positive patients (19). However, the ORR of anti-PD monotherapy was not as satisfactory as expected, around 11% as reported from the ATTRACTON and KEYNOTE studies (19, 33). TIME of advanced gastric cancer characterized by T cell exclusion or infiltration of immune-suppressive cells like tumor-associated macrophages were currently recognized as main causes (25). Thus different combination therapies targeting TIME with the aim to facilitate a synergistic effect are now under investigation (20, 21). Nevertheless, a promising conversion therapy with no doubt should integrate different therapeutic effects and obey scientific principles (21). The rationales underlying our combination therapy of SEEOX plus anti-PD-1 antibody could be summarized as follows. First, oxaliplatin has been reported to induce immunogenic cell death and increase tumor-specific effector T cell infiltration (29), hence the combination of anti-PD-1 antibody with oxaliplatin-based chemotherapies might induce an additive antitumor effect, which actually has been demonstrated in ATTRACTION-4 (34) and ORIENT-16 (35) studies, whereas, pembrolizumab plus cisplatin-based chemotherapy didn’t show survival benefit (23). Second, anti-PD therapy selectively modulate immunity and function in tumor microenvironment, while intra-arterial chemotherapy also administers cytotoxic drugs *via* tumor supplying blood vessels directly into the tumor region (28), which could increase the local drug concentration, generate a more inflammatory tumor environment, and consequently exacerbate the antitumor effect. Additionally, intra-arterial chemotherapy per se has displayed superior clinical benefits in our previous studies (26–28), and a multicenter phase III study initiated by our institution comparing SEEOX and SOX regimens in the neoadjuvant setting is under investigation (NCT02338518), interim analysis has demonstrated significant better ORR and survival outcome in the intra-arterial chemotherapy group. Besides, as most untreated gastric cancer was T cell exclusion (20, 25), it is safe to apply intra-arterial chemotherapy, with little risk of destroying infiltrated tumor-specific effector T cells at the beginning. Overall, our combination of intra-arterial chemotherapy and anti-PD-1 antibody in conversion therapy meets the basic principle of immune checkpoint inhibitors, and the excellent ORR (77.8%) was the best proof. Meanwhile, the remarkable pathological response (19/30, 63.3%) and high pathologically complete remission rate (7/30, 23.3%) also revealed the effect of the combinational regimen in our study.

Furthermore, we comprehensively dissected the TIME before and after intra-arterial chemotherapy in order to uncover possible mechanisms underlying the additive effects. According to Prof. Chen, TIME was classified into four different types based on the expression of PD-L1 (B7-H1) and the infiltration of TILs in tumor microenvironment (21). PD-L1-positive tumors with TILs infiltration (Type II) were considered the optimized candidates for anti-PD therapies. Therefore, to benefit from the anti-PD therapy, increasing TILs trafficking are essential and may be the first step for many ‘immunological ignorant or resistant’ tumors. Here in our study, we found intra-arterial SEEOX induced significant increasement in the number of TLSs (P = 0.004). TLSs are ectopic lymphoid organs formed in non-lymphoid tissues, set in the most front for adaptive immune response, usually accompanied with severe or persistent inflammation (36). Functionally similar to secondary lymph organs, TLSs provide privileged sites for nearby tumor antigen presentation, T and B cell differentiation and proliferation, consequently promote effector cells migration to the tumor site, which has been demonstrated to predict a favorable pathological response to immune checkpoint inhibitors (ICIs) and was usually related to a beneficial prognosis (36, 37). Besides, it was also reported TLS density correlates with CD4^+^ and CD8^+^ T cells in tumors (38), which is partly consistent with our study. The percentage of CD8^+^ T cells was significantly increased after chemotherapy, while CD4^+^ T cells didn’t show significant difference, and conversely the percentage of CD3^+^ cells (total of CD4^+^ and CD8^+^ T cells) greatly decreased. Since we only collected tumor samples before treatment and surgical specimen after 3 cycles of chemotherapies, the non-specific cytotoxic effect of chemotherapy on immune cells might be a possible explanation. Meanwhile, we detected increased expression of PD-1 in tumor and immune cells, which possibly represented the treatment-induced escape mechanisms counteracting the chemotherapy. Thus subsequent anti-PD-1 antibody was an appropriate method to unleash and sustain the anti-tumor effect. We also observed phenotype shifting of TAMs from immune-suppressive CD68^+^CD163^+^ TAM2 to pro-inflammatory/anti-tumor CD68^+^CD163^-^TAM1. TAM2 has long been recognized to be associated with tumor progression, metastasis and poor prognosis, and recent evidence revealed TAM2 suppressed the effect of ICIs *via* secreting anti-inflammatory cytokines and exosomes, and also increasing superficial PD-L1 (39). So the phenotype transition driven by intra-arterial chemotherapy could also enhance the efficacy of anti-PD therapies. As a result, intra-arterial chemotherapy is a potent tumor microenvironment manipulating method which favors the response to ICIs, meanwhile sequential ICIs sustain the immunogenic environment, then exacerbating anti-tumor effect.

In addition, our data preliminarily indicated that patients who continued with anti-PD-1 antibody after surgery exhibited significantly longer OS and PFS than those who only received standard SOX chemotherapy (**Figures 3E, F**). However, because of the limited case number, further investigation was warranted to confirm the conclusion, and maybe more convinced results could be expected from the undergoing phase III ATTRACTION-5 (NCT03006705) study, investigating the efficacy of nivolumab in combination with standard chemotherapy during adjuvant treatment period.

The addition of anti-PD-1 antibody did not substantially affected the safety profile of intra-arterial chemotherapy. The grade 3 AEs were encountered in 19.4% patients with no grade 4 AEs observed, which is similar to our previous study with SEEOX only (28). The rate of AEs associated with anti-PD-1 was low, only 2 patients experienced dermatitis or diarrhea, and no severe AEs occurred. No patient died of surgery, two patients had anastomotic fistula and another experience hemorrhage, all were controlled with conservative treatment. Hence 3 cycles of SEEOX plus anti-PD-1 antibody was well tolerated and did not increase the following surgical morbidity.

Due to the limited follow-up period of 19.7 months, the median OS of all the patients was not reached, but the mean OS was 18.5 months (95% CI 16.5-20.6) at the cut-off date March 2022; most R0 patients were alive, and long-term survival benefit has already been confirmed in this population (8–10, 12), so obviously intra-arterial chemotherapy with sequential anti-PD-1 in conversion therapy has improved the survival time of patients with unresectable gastric cancer, which was around one year as reported for palliative chemotherapies (4–6). By the way, the follow-up of the study is ongoing, and we will report the long-term survival outcomes in the future.

Of note, there lacks precise definition for unresectable gastric cancer. Yoshida classified advanced stage IV GC into 4 categories (12). Based on this classification, most of our cases should belong to category 1 and 2. As they expected most responders to the induction therapy were from category 2, patients in our cohort were just appropriate for the examination of the effect of conversion therapies.

There are some limitations to our study. First, the sample size is relatively small and there is no control group. However we enrolled all patients who received SEEOX plus anti-PD-1 antibody between May 2019 and October 2020 in our institution, which partly reduced the selection bias, but solid conclusions could only be drawn with further prospective controlled studies. Second, we only analyzed TIME before treatment and after 3 cycles of SEEOX, didn’t dissect the TIME in serial tissues among the process of treatment, so dynamic immune response might be missed, whereas, in the human study, sequential biopsy was not highly possible. Besides, we should recognize that the TIME is a complex system involving kinds of immune cells and cytokines, and more work is needed to decipher underlying mechanisms.

In conclusion, our study for the first time proved that intra-arterial chemotherapy with sequential anti-PD-1 antibody is a safe and effective conversion treatment strategy for unresectable GC, and preliminarily revealed that TIME transformation induced by intra-arterial chemotherapy including the formation of TLSs, infiltration of CD8^+^ T cells and phenotype transition of TAM might possibly mediate the additive and synergistic effect of the combination regimen.

## Data availability statement

The original contributions presented in the study are included in the article/**Supplementary Material**. Further inquiries can be directed to the corresponding author.

## Ethics statement

The studies involving human participants were reviewed and approved by Ethics Committee of Jinling Hospital, School of Medicine, Nanjing University. The patients/participants provided their written informed consent to participate in this study.

## Author contributions

XX, FG, and GL conceived and designed the study, analysed and interpreted the data, and wrote the manuscript. XX and LM contacted patients and collected the specimen. LM, XZ, and ZA recorded the clinical data. JL, JZ, and MH performed multiplex immunofluorescence and did TIME analysis. All authors read and approved the final version of the manuscript.
