# Supplementary material for: Efficacy of intra-arterial chemotherapy with sequential anti-PD-1 antibody in unresectable gastric cancer: A retrospective real-world study
Source: Front Oncol. 2023 Jan 5;12:1015962. doi: 10.3389/fonc.2022.1015962 (PMC9849699; doi:10.3389/fonc.2022.1015962)
Supplement: Supplementary file 1 [file Table_1.docx]

Supplementary Material

Table S1. Details of unresectable factors in each patient

| Case | Bulky lymph nodes | Invasion of adjacent organs | Large distal tumor affecting duodenum closure | Peritoneal metastasis | Distant lymph node metastasis | Distant organ metastasis |
| --- | --- | --- | --- | --- | --- | --- |
| 1 |  | + |  |  | + |  |
| 2 |  |  | + |  |  |  |
| 3 |  | + |  |  |  |  |
| 4 |  | + |  |  |  |  |
| 5 |  |  |  |  | + |  |
| 6 |  |  |  |  | + | Descending colon |
| 7 | + |  |  |  | + |  |
| 8 |  |  |  |  | + |  |
| 9 |  |  |  |  | + |  |
| 10 | + |  |  |  | + |  |
| 11 |  |  | + |  |  |  |
| 12 |  |  |  |  | + |  |
| 13 |  |  |  |  | + | Liver |
| 14 |  |  |  | + |  |  |
| 15 |  |  | + |  |  |  |
| 16 |  |  | + |  |  |  |
| 17 | + |  |  |  | + |  |
| 18 |  |  |  |  | + | Ovary |
| 19 | + |  |  |  |  |  |
| 20 |  |  |  |  |  | Liver |
| 21 |  |  |  | + |  |  |
| 22 | + |  |  |  |  |  |
| 23 |  |  |  |  | + |  |
| 24 |  |  |  | + |  |  |
| 25 | + |  |  |  |  |  |
| 26 |  | + |  |  |  |  |
| 27 |  |  |  | + |  |  |
| 28 | + |  |  |  |  |  |
| 29 |  | + | + |  |  |  |
| 30 | + |  |  |  |  |  |
| 31 | + |  |  |  | + |  |
| 32 | + |  |  |  | + |  |
| 33 | + |  |  |  |  |  |
| 34 | + |  |  |  |  |  |
| 35 | + |  |  |  |  |  |
| 36 | + |  |  |  |  |  |

Table S2. Demographic, oncological and therapeutic information of patients for tumor immunity in the microenvironment analysis

| **Variable** | **Patients (N=7)** |
| --- | --- |
| Age (years), mean (range) | 61 (52–73) |
| Sex, N (%) |  |
| Male | 6 (85.7%) |
| Female | 1 (14.3%) |
| Tumor Location, N (%) |  |
| Upper third | 5 (71.4%) |
| Diffuse or undefined | 2 (28.6%) |
| Borrmann Type, N (%) |  |
| II | 2 (28.6%) |
| III | 3 (42.9%) |
| Large III（> 8 cm） | 1 (14.3%) |
| IV | 1 (14.3%) |
| Tumor invasion^*^, N (%) |  |
| cT3 | 1 (14.3%) |
| cT4a | 6 (85.7%) |
| Lymph node metastasis^*^, N (%) |  |
| cN1 | 4 (57.1%) |
| cN2 | 1 (14.3%) |
| cN3 | 2 (28.6%) |
| TNM Stage^*^, N (%) |  |
| cIII | 7 (100%) |
| Clinical response per RECIST guidelines (version 1.1), N (%) |  |
| Partial response | 5 (71.4%) |
| Stable disease | 2 (28.6%) |
| Type of resection, N (%) |  |
| Total gastrectomy | 7 (100%) |
| R0/R1 resection | 6/1 |
| ypT staging, N (%) |  |
| T1b | 1 (14.3%) |
| T3 | 4 (57.1%) |
| T4a | 2 (28.6%) |
| ypN staging, N (%) |  |
| N0 | 3 (42.9) |
| N2 | 2 (28.6%) |
| N3a | 1 (14.3%) |
| N3b | 1 (14.3%) |
| ypTNM, N (%) |  |
| IA | 1 (14.3%) |
| IIA | 1 (14.3%) |
| IIB | 1 (14.3%) |
| IIIA | 2 (28.6%) |
| IIIB | 1 (14.3%) |
| IIIC | 1 (14.3%) |
| TRG, N (%) |  |
| TRG1 | 1 (14.3%) |
| TRG2 | 3 (42.9%) |
| TRG3 | 3 (42.9%) |

^*^ In accordance with the AJCC eight edition gastric cancer TNM staging system
